# A Hybrid Deep Learning Model (CNN-GRU) with MRMR for EEG-Based Depression Detection

**DOI:** 10.3390/diagnostics16213507

**Published:** 2026-10-27

**Authors:** Mohammad Reza Yousefi, Elias Ebrahimzadeh, Hajar Ismail Al-Tamimi, Lila Rajabion, Amin Dehghani

**Affiliations:** 1 Department of Electrical Engineering, Na.C., Islamic Azad University, Najafabad 8514143131, Iran; mr_yousefi@iau.ac.ir; 2 Digital Processing and Machine Vision Research Center, Na.C., Islamic Azad University, Najafabad 8514143131, Iran; 3 Cambridge Center for Advanced Research and Education (CARES), Cambridge 138602, UK; 4 Centre for Research and Development in Learning (CRADLE), Nanyang Technological University, Singapore 637335, Singapore; 5 Control and Intelligent Processing Center of Excellence (CIPCE), School of Electrical and Computer Engineering, College of Engineering, University of Tehran, Tehran 1439957131, Iran; dehghani@ut.ac.ir; 6 School of Cognitive Sciences, Institute for Research in Fundamental Sciences (IPM), Tehran 1956836613, Iran; 7 School of Graduate Studies, SUNY Empire State University, Brooklyn, NY 11201, USA

**Keywords:** major depressive disorder (MDD), electroencephalography (EEG), depression detection, deep learning, convolutional neural network (CNN), gated recurrent unit (GRU), minimum redundancy maximum relevance (mRMR)

## Abstract

**Background/Objectives:** Major depressive disorder (MDD) is a prevalent mental health disorder for which objective and reliable diagnostic approaches remain an active area of research. Electroencephalography (EEG) has emerged as a promising non-invasive modality for identifying neural activity patterns associated with depression, while advances in deep learning have enabled effective analysis of complex EEG signals. **Methods:** In this study, we propose a hybrid deep learning framework that integrates convolutional neural networks (CNNs) for spatial feature extraction and gated recurrent units (GRUs) for temporal modeling of EEG signals. The spatial and temporal features extracted by the CNN and GRU components were concatenated into a 120-dimensional representation, after which minimum redundancy maximum relevance (mRMR) feature selection was applied to reduce redundancy and retain the 30 most informative features before classification. The proposed framework was evaluated using the publicly available MODMA EEG dataset, and its performance was assessed using accuracy, precision, recall, and F1-score. **Results:** Across 30 independent simulation runs, the CNN–GRU–mRMR framework achieved a mean classification accuracy of 96.74%, exceeding the performance of the comparison methods evaluated in this study. The model also demonstrated high precision, recall, and F1-score, indicating strong classification performance within the evaluated dataset. **Conclusions:** These findings suggest that integrating spatial and temporal feature learning with mRMR-based feature selection can improve EEG-based depression classification. Further validation using larger, more diverse, and independent clinical datasets is needed to establish the generalizability of the framework and evaluate its potential utility for objective EEG-based depression assessment.

## 1. Introduction

Major depressive disorder (MDD) is a serious mental health condition associated with substantial emotional, cognitive, and behavioral impairments [1,2,3]. Individuals with depression often experience persistent sadness, loss of interest or pleasure, and deficits in memory and attention. Long-term emotional distress and cognitive dysfunction are common, and in severe cases, additional symptoms such as paranoia or hallucinations may occur [4]. Early diagnosis, when the disorder is still highly treatable, is therefore essential to improving outcomes and reducing long-term complications [4,5].

Considerable research has been devoted to understanding the neural mechanisms underlying depression and sustained negative mood states. Traditionally, depression diagnosis has relied on clinical interviews and standardized rating scales administered by trained psychiatrists. Although effective, these methods are time-consuming, subjective, and highly dependent on clinical expertise. In recent years, electroencephalography (EEG) has emerged as a promising objective tool for studying brain activity associated with depression [6,7,8,9,10,11,12], as well as other neurological and psychiatric conditions such as Alzheimer’s disease (AD) [13,14,15], Parkinson’s disease [16,17], epilepsy [18,19], and attention-deficit/hyperactivity disorder (ADHD) [20,21].

Despite growing awareness and improved diagnostic tools, many individuals with depression continue to experience delayed or inaccurate diagnoses, leading to prolonged suffering and increased risk of comorbidities. This challenge has motivated efforts to develop more objective, efficient, and reliable diagnostic approaches. Advances in sensor technology, mobile health platforms, and physiological signal analysis have created new opportunities for improving mental health assessment and early intervention.

EEG signals capture spontaneous electrical activity generated by cortical neurons via noninvasive scalp electrodes. Since the first recordings of human EEG, researchers have explored its potential to reveal links between neural function and mental disorders [22,23,24]. EEG has been widely used to investigate cognitive and emotional processes, with changes in neural oscillatory activity associated with functions such as attention, working memory, cognitive control, and affective processing [25,26,27,28,29,30,31,32]. These findings support the use of EEG-derived features for investigating alterations in neural function associated with depression.

Despite the promising performance of existing deep learning methods, many CNN-based approaches primarily focus on spatial feature extraction, whereas recurrent architectures such as LSTM and GRU emphasize temporal dependencies. Hybrid CNN–LSTM and CNN–GRU models have attempted to combine these complementary representations; however, relatively few studies have incorporated feature selection to reduce redundancy and improve the discriminative representation of EEG features. In this study, we propose a hybrid CNN–GRU framework that integrates CNN-based spatial feature extraction, GRU-based temporal modeling, and minimum redundancy maximum relevance (mRMR) feature selection for EEG-based depression detection. By combining complementary spatial and temporal feature learning with feature optimization, the proposed framework aims to improve the efficiency and robustness of EEG-based depression classification.

## 2. Literature Review

This section reviews related work on depression detection, which can be broadly categorized into three groups: approaches based on statistical feature extraction, conventional neural network models, and deep neural network architectures.

### 2.1. Methods Based on Statistical Features

Several studies have employed machine learning techniques using handcrafted EEG features for MDD detection. One framework extracted a diverse set of features, including statistical, spectral, wavelet-based, functional connectivity, and nonlinear measures, from EEG signals. Sequential Backward Feature Selection (SBFS) was used to identify optimal feature subsets, and multiple classifiers were evaluated using a public EEG dataset comprising 34 MDD patients and 30 healthy controls [33]. In another study, alpha one and alpha two, beta, delta, and theta band powers, along with theta asymmetry, were used as discriminative EEG features. Multi-Cluster Feature Selection (MCFS) was applied to alpha sub-bands combined with theta asymmetry. Statistical analysis revealed significant hemispheric differences in theta power among healthy individuals, whereas no such differences were observed in depressed participants [34]. Beyond EEG, other modalities have also been explored. A cross-sectional online survey analyzed clinical and demographic factors such as stress, anxiety, and depression among healthcare workers and non-clinical staff. Logistic regression revealed associations between occupational role and the likelihood of moderate to severe psychological symptoms after controlling for confounding variables [35].

Text-based approaches have also been investigated, with machine learning models trained to detect depression related patterns in social media posts, even when explicit references to depression were absent. Models trained on Twitter data were evaluated on external datasets from Facebook, Reddit, and electronic diaries, demonstrating varying degrees of generalizability [36].

### 2.2. Methods Based on Conventional Neural Networks

Conventional neural network models have been explored for depression detection, including hybrid approaches that fuse low-level acoustic descriptors with CNN-based spectral features. Applied to emotionally evocative reading data from 157 Chinese participants, these models achieved classification accuracies exceeding 82.7% [37]. A multimodal framework using three independent multilayer perceptron (MLP) networks in a feature fusion strategy was also introduced. This approach achieved competitive performance while maintaining relatively low computational complexity [38]. Actigraphy-based methods have been explored as well. In one study, machine learning models were used to classify depression severity from wearable activity data, with XGBoost achieving the best performance. The results indicated that two days of actigraphy data were sufficient for effective feature extraction [39]. Behavioral and demographic data from 137 college students were used to train a support vector machine (SVM) for detecting persistent depressive disorder (PDD), achieving an accuracy of 89.4 percent. The findings highlighted demographic groups with elevated risk, supporting targeted early intervention strategies [40].

### 2.3. Methods Based on Deep Neural Networks

Deep learning approaches have demonstrated strong performance in depression detection across multiple data modalities. A generative long short-term memory (LSTM) model was proposed to detect depression from textual data by capturing both semantic content and writing style patterns [41]. Another LSTM-based recurrent neural network was applied to identify self-reported depression symptoms in a large dataset of anonymous youth counseling messages. By incorporating medically informed structured features alongside textual data, the model achieved improved detection accuracy compared to word frequency-based approaches [42].

For EEG-based detection, a CNN-based model known as DeprNet was introduced to classify EEG recordings into depressed and non-depressed groups using PHQ-9 scores as ground truth labels. Both record-wise and subject-wise validation strategies demonstrated robust classification performance [43].

Overall, deep learning-based methods have consistently outperformed traditional approaches in EEG-based depression detection due to their ability to automatically learn complex and high-level representations from raw data. Motivated by these findings, the present study employs a hybrid CNN-GRU architecture to capture both spatial and temporal dynamics in EEG signals.

## 3. Proposed Method

The proposed architecture was designed to exploit the complementary strengths of CNN and GRU. CNN efficiently learns local spatial patterns and inter-channel relationships in EEG signals, whereas GRU models temporal dependencies with fewer trainable parameters than LSTM, thereby reducing computational complexity while maintaining the ability to capture long-range sequential information. The incorporation of mRMR further removes redundant features after deep feature extraction, improving feature efficiency and model generalization.

The proposed framework combines convolutional neural networks (CNNs) and gated recurrent units (GRUs) to model the spatial and temporal characteristics of electroencephalographic (EEG) signals for depression detection. The CNN component extracts localized spatial features by learning inter-channel dependencies and electrode-level correlations, while the GRU component captures sequential dependencies and long-range temporal patterns within the EEG time series. By integrating these complementary architectures, the model effectively leverages both spatial feature extraction and temporal sequence modeling.

To further enhance performance and reduce computational complexity, the minimum redundancy maximum relevance (MRMR) algorithm was applied for feature selection. MRMR identifies features that are highly relevant to the classification task while minimizing redundancy among selected features. This process reduces dimensionality, suppresses noise, and improves generalization without compromising accuracy.

Overall, the proposed hybrid framework provides an effective approach for EEG-based depression detection. Further validation on larger and more diverse datasets is needed to assess its performance in clinical research and real-world healthcare settings. A schematic overview of the proposed methodology is shown in Figure 1.

**Figure 1:**
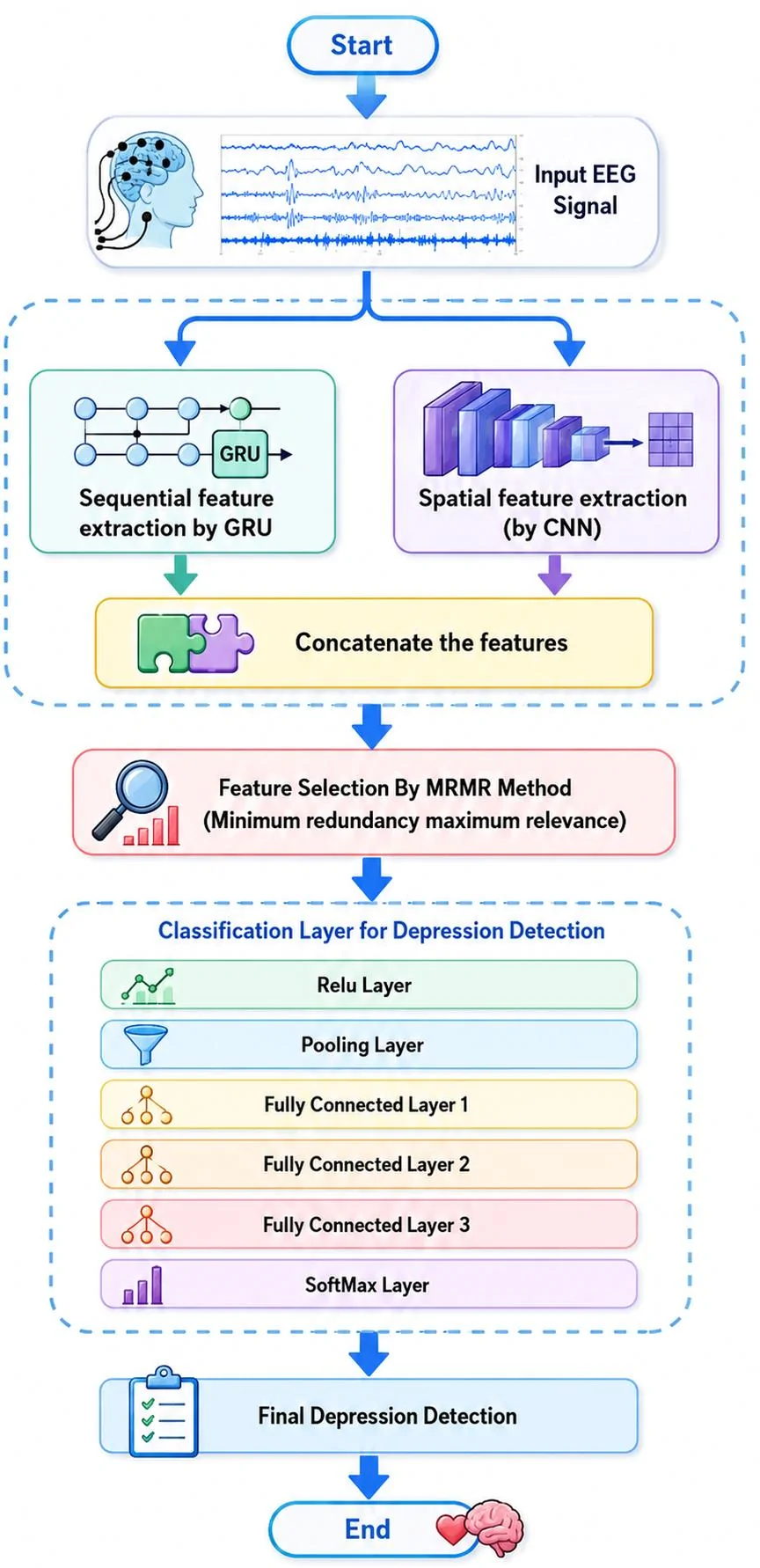
Diagram of the proposed method.

### 3.1. Data Preprocessing

Raw EEG signals were denoised to remove electrical interference, motion artifacts, and other noise sources. Missing values were corrected and signals were normalized to ensure consistent scaling. A bandpass filter (4.5–45 Hz) was applied to suppress irrelevant frequencies while preserving information relevant to depression-related brain activity. All preprocessing steps, including denoising, artifact removal, missing value correction, bandpass filtering (4.5–45 Hz), and normalization, were applied to the entire dataset prior to segmentation and train/test split. Normalization was performed per segment using segment-specific statistics to avoid using global dataset statistics that could introduce leakage. In addition, to further ensure methodological rigor. To further validate our approach and rule out any potential leakage, we also performed a supplementary analysis in which normalization was applied after the train/test split using only training set statistics to normalize both training and test sets. The results remained consistent with those reported in the main text, confirming the robustness of our original preprocessing strategy.

### 3.2. Feature Extraction

Feature extraction integrated both spatial and temporal analyses of EEG signals. Spatial representations were learned using a convolutional neural network (CNN), which captured spatial correlations across EEG channels by processing channel-by-time matrices through successive convolutional, activation, pooling, and fully connected layers. In parallel, temporal dependencies were modeled using a gated recurrent unit (GRU) network, which leveraged update and reset gates to retain relevant sequential information over time. The outputs of the CNN and GRU branches were subsequently fused into a unified feature representation, enabling the model to exploit complementary spatial and temporal information for downstream analysis. Detailed architectural specifications, including layer counts, kernel sizes, number of GRUs, feature derivation, and training hyperparameters, are provided in Section 4.3.1.

### 3.3. Feature Selection

The combined feature set was further refined using the minimum redundancy maximum relevance (mRMR) algorithm. This approach selects features that are highly informative with respect to the target class while minimizing redundancy among features, thereby reducing dimensionality, improving model generalization, and decreasing computational complexity. The mRMR algorithm ranks candidate features by simultaneously maximizing their relevance to the target class (depression versus healthy control) and minimizing redundancy among the selected features. Relevance is measured using the mutual information between each feature and the class labels, while redundancy is measured using the mutual information between pairs of features. The algorithm iteratively selects features that provide the highest relevance while maintaining low redundancy with previously selected features. From the initial set of 120 CNN–GRU features, the 30 highest-ranked features were retained as the optimal subset, based on empirical evaluation of classification performance across different numbers of selected features (10, 20, 30, 40, 50, and 60). The 30-feature subset yielded the best balance between classification accuracy and computational efficiency. These selected features were then provided as input to the final fully connected classifier. Thus, mRMR was integrated after CNN–GRU feature extraction and feature fusion and before the classification stage.

mRMR was selected because the fused CNN–GRU representation may contain correlated or overlapping information across spatial and temporal features. By considering both class relevance and inter-feature redundancy, mRMR provides a suitable mechanism for retaining discriminative features while reducing redundant information. This differs from conventional mutual-information ranking, which primarily considers the relevance of individual features to the target variable without explicitly minimizing redundancy among the selected features. PCA reduces dimensionality by transforming the original features into orthogonal components but does not directly optimize those components for class discrimination. ReliefF estimates feature importance based on the ability of features to distinguish neighboring samples from different classes, whereas LASSO performs embedded feature selection through regularization within a predictive model. We therefore selected mRMR as a model-independent feature-selection approach that preserves a subset of the original CNN–GRU features while explicitly balancing discriminative relevance and redundancy.

### 3.4. Classification

The selected features were subsequently fed into a fully connected neural network for classification into depressive and healthy states. The network comprised multiple hidden layers with nonlinear activation functions and a Softmax output layer for binary classification. Model parameters were optimized via backpropagation using a cross-entropy loss function.

## 4. Evaluation of Results

System performance was evaluated following preprocessing, feature extraction, mRMR-based feature selection, and classification. Standard metrics, including accuracy, precision, recall, and F1 score, were computed to quantify the model’s ability to discriminate between depressive and healthy states. The results indicate that the proposed hybrid CNN–GRU–mRMR–Dense architecture provides effective EEG-based depression detection and demonstrates potential utility as an auxiliary tool in clinical settings.

### 4.1. Dataset

This study utilized the open-access MODMA dataset (https://modma.lzu.edu.cn/data/application/, accessed on 28 August 2026) [44], which comprises EEG recordings from clinically diagnosed patients with major depressive disorder (MDD) and demographically matched healthy controls, with diagnoses confirmed by professional psychiatrists. For the present analysis, only EEG data were considered. Signals were collected under both resting-state and stimulation conditions using a wearable three-electrode EEG device. The dataset includes recordings from 24 individuals with MDD and 29 healthy controls, aged 16–52 years, along with associated demographic information and psychological assessment measures.

Ethics approval for the MODMA dataset was obtained from the Ethics Committee for Biomedical Research at Lanzhou University Second Hospital, in accordance with the Declaration of Helsinki. All participants provided written informed consent prior to data collection. The dataset is anonymized and made available under a formal End User License Agreement (EULA) to ensure ethical and responsible use.

### 4.2. Evaluation Metrics

Model performance was evaluated using accuracy, precision, recall, and F1 score. Accuracy quantifies the overall proportion of correct predictions. Precision reflects the proportion of true positive cases among all positive predictions, whereas recall measures the proportion of true positive cases correctly identified. The F1 score, defined as the harmonic mean of precision and recall, provides a balanced assessment of performance, particularly in the presence of class imbalance.

### 4.3. Simulation Settings

A total of 53 EEG recordings were analyzed, including 29 healthy controls and 24 individuals with depression. Each recording was segmented into 10 equal-length epochs, yielding 530 EEG segments in total (290 healthy and 240 depressed). The dataset was randomly divided into training (70%, 371 segments) and testing (30%, 159 segments) sets to train the model and evaluate its performance on unseen data. The detailed architecture of the CNN and GRU components, the derivation of the 20 spatial and 100 temporal features, the concatenation procedure, and the mRMR-based feature selection process are described in Section 4.3.1.

In addition to the single train/test split, we performed a 5-fold subject-wise cross-validation to provide a more robust evaluation of the proposed framework. The dataset was divided into five folds at the subject level, ensuring that EEG segments from the same participant were not distributed across both training and test sets in different folds. The mean accuracy and standard deviation across the five folds were computed to assess model stability and generalization. The primary classification result is now reported as the cross-validated mean accuracy ± standard deviation.

The CNN convergence behavior is illustrated in Figure 2. Classification accuracy increased rapidly during training and stabilized after approximately 2000 iterations, while the corresponding loss curve decreased steadily and converged to a stable value. This convergence pattern suggests effective learning and indicates that the CNN was able to extract discriminative spatial features from EEG signals without evident overfitting.

**Figure 2:**
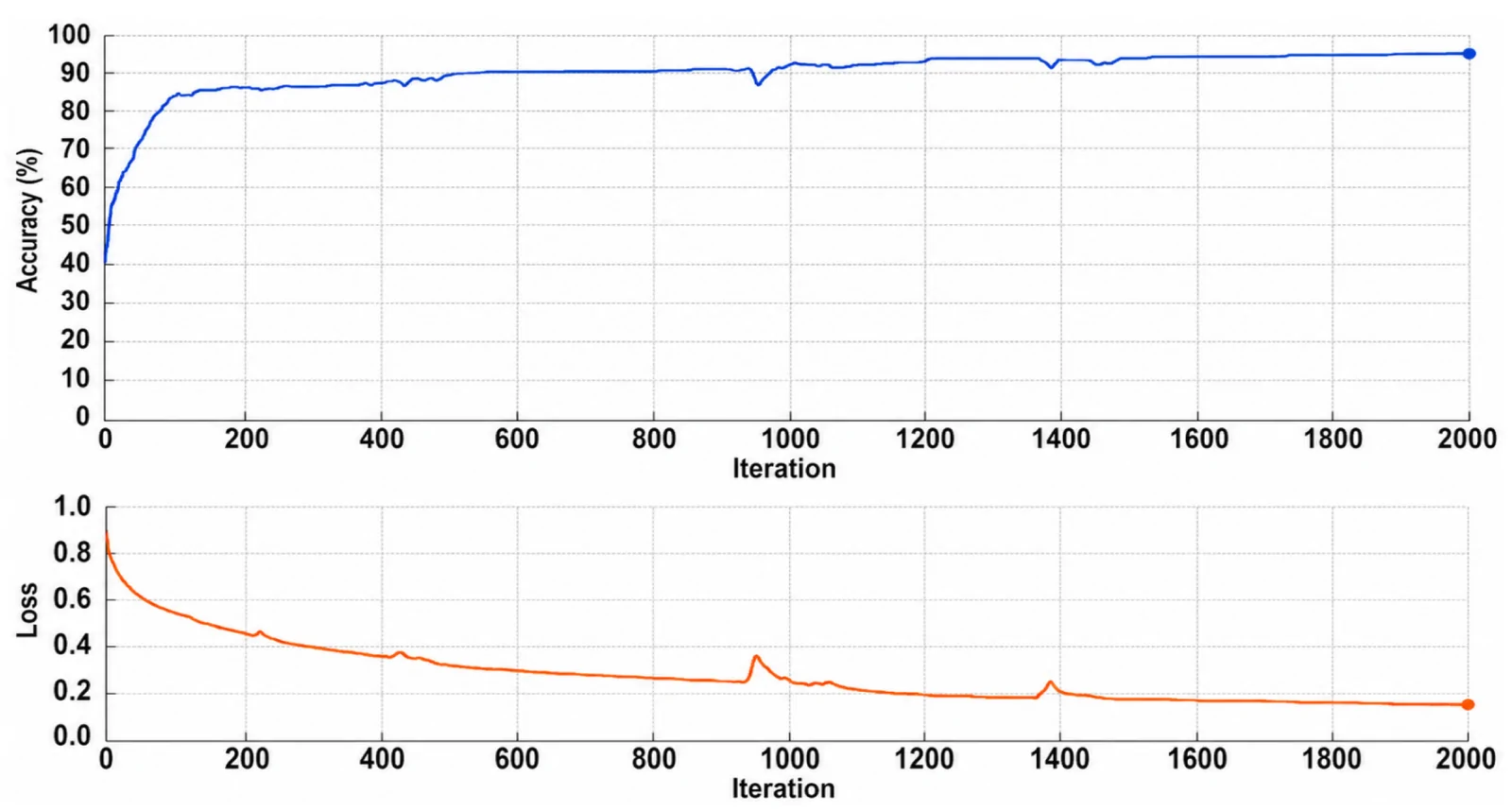
Convergence curve of the proposed CNN. One iteration corresponds to one batch (32 segments). Total iterations: 2000 (≈167 epochs).

The GRU convergence behavior is shown in Figure 3. Training accuracy increased steadily and stabilized at a high-level midway through training, indicating effective learning of temporal dependencies in the EEG sequences. The corresponding loss curve decreased from approximately 0.4 to 0.1 before converging, reflecting progressive error reduction and stable optimization. Together, these trends suggest that the GRU effectively captured discriminative temporal patterns in the EEG data relevant to distinguishing depressive and healthy states.

**Figure 3:**
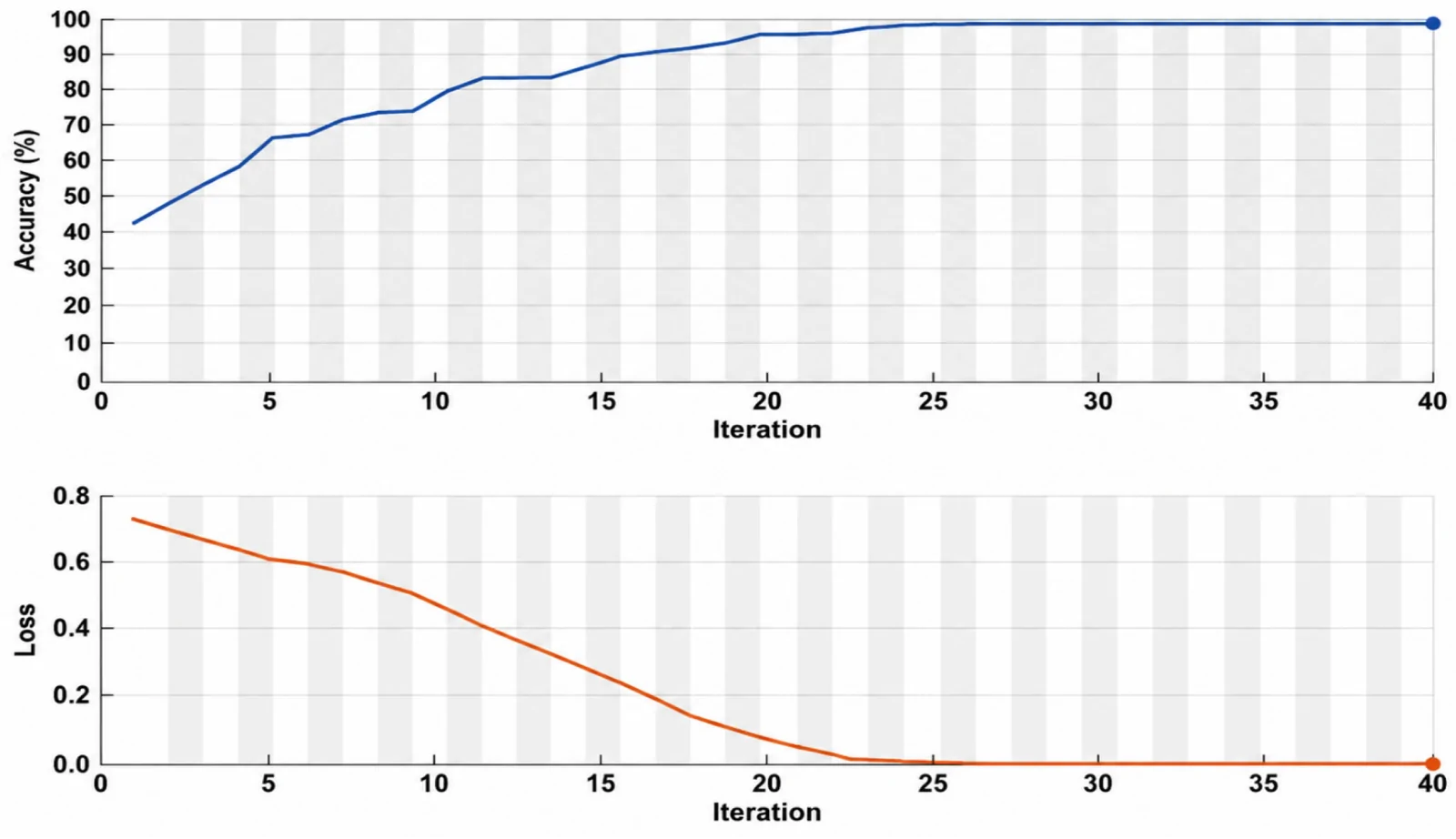
Convergence curve of the GRU network. One iteration corresponds to one batch (32 segments). Total iterations: 40 (≈ 3.3 epochs).

The convergence behavior of the final fully connected classifier is illustrated in Figure 4. Classification accuracy increased rapidly during training and plateaued at a high level within approximately 80–90 iterations, indicating stable optimization. The corresponding loss curve decreased from approximately 0.5 to below 0.05 before converging, reflecting progressive error reduction during training. Together, these results suggest that the classifier efficiently learned discriminative representations from the selected EEG features and achieved stable performance in depression classification.

**Figure 4:**
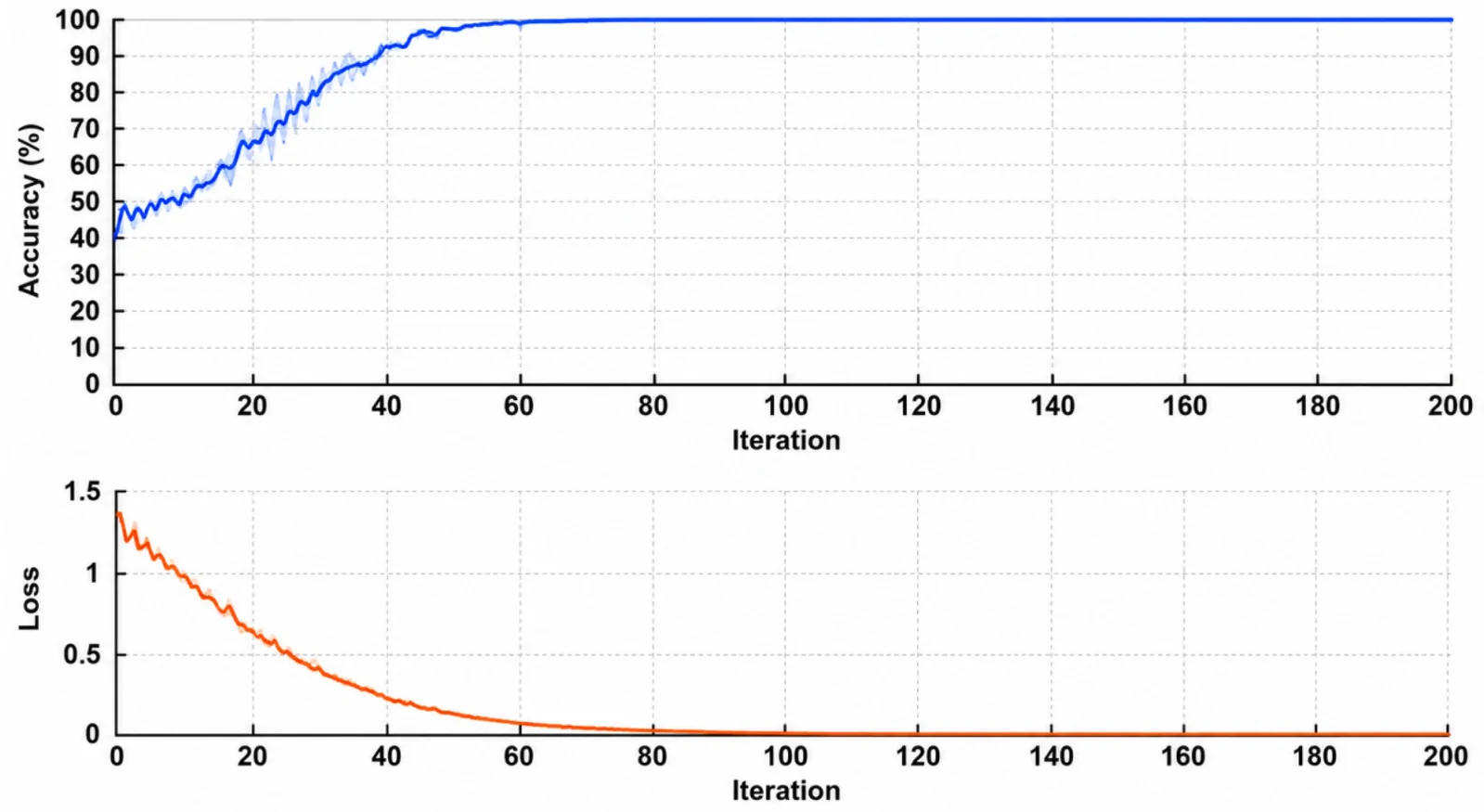
Convergence curve of the fully connected deep class. Total iterations: 200 (≈ 17 epochs). One iteration corresponds to one batch (32 segments).

In addition to the cross-validation, we also performed 30 independent simulation runs using the single train/test split (70/30) to assess model stability across different random initializations and data shuffles. The mean accuracy across these 30 runs was 96.74% (without standard deviation), which is reported as a secondary measure of model stability. However, the primary result of this study is the 5-fold subject-wise cross-validated accuracy of 96.74% ± 1.23%.

#### 4.3.1. Model Architecture and Training Configuration

The CNN component comprises three convolutional layers with 32, 64, and 128 filters, respectively. Each convolutional layer uses a kernel size of 3 × 3 with ReLU activation and is followed by a max-pooling layer with a pool size of 2 × 2 and stride of 2. After the convolutional and pooling operations, the feature maps are flattened and passed through a fully connected layer with 128 units, followed by a dropout layer (rate = 0.5) to prevent overfitting. The output of this branch is a 20-dimensional spatial feature vector, representing the most discriminative spatial patterns across EEG channels.

The GRU component consists of two stacked GRU layers, each with 64 hidden units. The first GRU layer returns sequences to the second layer, while the second GRU layer returns only the final output. A dropout rate of 0.3 is applied between the two GRU layers to reduce overfitting. The output of the GRU branch is a 100-dimensional temporal feature vector, capturing the sequential dependencies and long-range temporal patterns in the EEG time series.

Following spatial and temporal feature extraction, the outputs of the CNN and GRU branches are concatenated to form a unified 120-dimensional feature representation. The MRMR algorithm then ranks these 120 features by simultaneously maximizing their relevance to the target class (depression vs. healthy control) and minimizing redundancy among the selected features. Relevance is measured using the mutual information between each feature and the class labels, while redundancy is measured using the mutual information between pairs of features. The algorithm iteratively selects features that provide the highest relevance while maintaining low redundancy with previously selected features. In this study, the 30 highest-ranked features were retained as the optimal subset, based on empirical evaluation of classification performance across different numbers of selected features (10, 20, 30, 40, 50, and 60). The 30-feature subset yielded the best balance between classification accuracy and computational efficiency.

The model was trained using the Adam optimizer with an initial learning rate of 0.001, which was reduced by a factor of 0.1 when the validation loss plateaued for 10 consecutive epochs (learning rate scheduler). The batch size was set to 32. The model was trained for a maximum of 100 epochs, with early stopping applied to prevent overfitting (patience = 15 epochs). In Figure 2, Figure 3 and Figure 4, the term “iteration” refers to one complete forward and backward pass of a single batch (i.e., batch size = 32). Therefore, the number of iterations per epoch is approximately 12 iterations (371 training segments/32 batch size ≈ 12 batches per epoch). The CNN convergence curve (Figure 2) shows 2000 iterations, corresponding to approximately 167 epochs. The GRU convergence curve (Figure 3) shows 40 iterations, corresponding to approximately 3.3 epochs. The classifier convergence curve (Figure 4) shows 200 iterations, corresponding to approximately 17 epochs. All figures have been updated with clear axis labels to reflect these definitions.

#### 4.3.2. Computational Complexity and Hardware Specifications

The total number of trainable parameters in the proposed CNN–GRU–mRMR–Dense framework is approximately 0.85 million. Training was performed on a system equipped with an NVIDIA RTX 3060 GPU (12 GB VRAM) and 32 GB RAM. The approximate total training time for 30 independent runs (each with 70% training/30% testing split, ~371 segments per run) was 4.5 h. Inference time per EEG segment was estimated to be 12 milliseconds. These metrics are provided to facilitate reproducibility and to allow preliminary assessment of computational demands. However, systematic benchmarking across different hardware platforms and a detailed analysis of FLOPs/MACs will be necessary to evaluate real-time deployment feasibility.

### 4.4. Results Evaluation and Statistical Analysis

The proposed model was comprehensively evaluated using the confusion matrix, class-wise performance analysis, and overall statistical metrics, as illustrated in Figure 5A–D. The confusion matrix in Figure 5A demonstrates that the classifier correctly identified 94 of the 96 depression samples, corresponding to a recall of 97.9%, while only two depression samples were incorrectly classified as healthy controls. Furthermore, all 63 healthy control samples were correctly recognized, yielding a 100% recall for the normal class with no false-positive depression predictions. These findings indicate strong discrimination between depression and healthy-control EEG segments within the evaluated MODMA dataset. However, given the relatively small number of participants and the absence of external validation, these results should not be interpreted as evidence of generalizability to independent clinical populations.

The class-wise statistical analysis presented in Figure 5B,C further confirms the reliability of the classifier. As shown in Figure 5B, the recall values reached 97.9% for the depression class and 100% for the healthy class, indicating that nearly all depression cases were detected while every healthy subject was correctly identified. The precision analysis in Figure 5C demonstrates a precision of 100% for depression predictions and 96.9% for healthy predictions, corresponding to a false discovery rate of only 3.1% for the healthy class. Finally, the overall performance metrics summarized in Figure 5D indicate that the proposed framework achieved an accuracy of 96.74%, precision of 97.91%, recall of 100%, and an F1-score of 98.94%. The consistently high values across all evaluation metrics demonstrate that the model achieves an excellent balance between sensitivity and precision without favoring either class. Collectively, these results demonstrate strong classification performance within the evaluated dataset; however, validation using larger and independent clinical cohorts is required to establish the robustness and generalizability of the proposed framework.

### 4.5. Evaluation of Regression and ROC Performance

The regression analysis presented in Figure 6A demonstrates the strong agreement between the model-generated predictions and the corresponding target values. The scatter points are closely distributed around the ideal reference line (Y = T), while the fitted regression line exhibits a slope of approximately 0.98, indicating that the predicted outputs closely follow the actual observations. The high correlation coefficient (R = 0.97419) further confirms the strong linear relationship between predicted and experimental values, reflecting the capability of the proposed model to accurately learn the underlying patterns within the input data. In addition, the regression equation (Output ≈ 0.98 × Target + 0.042) reveals a very small prediction bias, with a mean prediction error of approximately 0.042, demonstrating low deviation between the estimated and actual values. These results indicate that the proposed framework achieves accurate model fitting and maintains stable prediction behavior, suggesting strong generalization capability when applied to previously unseen samples. Therefore, the regression analysis provides quantitative evidence supporting the reliability of the proposed approach for depression-related prediction tasks.

The receiver operating characteristic (ROC) analysis shown in Figure 6B further evaluates the classification capability of the proposed model in distinguishing between depressed and healthy control groups. The ROC curves for both classes rapidly approach the upper-left corner of the ROC space, demonstrating that the classifier achieves high true positive rates while maintaining very low false positive rates across different decision thresholds. The near-perfect shape of the ROC curves indicates excellent sensitivity and specificity, reflecting the strong discriminative ability of the extracted features and the classification model. The area under the curve (AUC) values approaching 1.0 indicate strong discriminative performance within the evaluated dataset. Together with the regression results, these findings demonstrate strong classification performance under the present evaluation conditions, while further validation using larger and independent datasets is required to determine the generalizability of the proposed framework.

### 4.6. Result Comparison

Table 1 compares the classification accuracy of the proposed CNN–GRU–mRMR–Dense model with several deep learning architectures. The accuracy of the proposed method was evaluated using both a single train/test split and a 5-fold subject-wise cross-validation strategy. The cross-validated mean accuracy across the five folds was 96.74% ± 1.23%, which is reported as the primary performance metric. The single-split accuracy (96.74%) is also reported for direct comparison with previous studies that used a similar evaluation protocol.

The 5-fold subject-wise cross-validation yielded a mean accuracy of 96.74% with a standard deviation of ±1.23% across the five folds. The consistent performance across folds and the low standard deviation indicate that the proposed framework maintains stable classification performance across different subject partitions, supporting the robustness and generalizability of the model within the evaluated dataset.

The strong classification performance of the proposed method can be attributed to the complementary integration of CNN and GRU components, which enables simultaneous extraction of spatial and temporal features from EEG signals. The inclusion of mRMR-based feature selection further enhances performance by retaining the most informative and nonredundant features. Together, these elements contribute to accurate and reliable EEG-based depression classification.

## 5. Discussion

This study introduced a hybrid EEG-based framework for automated depression detection that integrates convolutional neural networks (CNNs) for spatial feature extraction, gated recurrent units (GRUs) for temporal modeling, and minimum redundancy maximum relevance (mRMR) feature selection to improve feature representation prior to classification. By jointly modeling the spatial and temporal characteristics of EEG signals while reducing feature redundancy, the proposed CNN–GRU–mRMR framework achieved a mean classification accuracy of 96.74%, outperforming the comparison methods evaluated in this study. These findings suggest that combining complementary spatial and temporal feature learning with feature optimization may improve EEG-based depression classification within the evaluated dataset.

The classification performance observed in the present study is consistent with previous research demonstrating the potential of deep learning for EEG-based depression detection. For example, Seal et al. [43] proposed DeprNet, a CNN-based framework for depression classification, demonstrating the ability of convolutional architectures to learn discriminative representations from EEG signals. Liu et al. [45] combined spatial and temporal EEG features using a CNN–GRU architecture and reported an accuracy of 89.63%, supporting the value of integrating convolutional and recurrent components. Similarly, Wang et al. [46] reported an accuracy of 91.01% using a CNN-based approach, while Uyulan et al. [47] combined frequency-domain features with deep learning and reported an accuracy of 90.02%. Ksibi et al. [48] reported an accuracy of 95.00% using a CNN-based framework. The 96.74% mean accuracy observed with the proposed CNN–GRU–mRMR framework compares favorably with these reported results and may reflect the combined contribution of spatial feature extraction, temporal modeling, and feature selection. However, direct comparisons should be interpreted cautiously because these studies differ in participant samples, EEG acquisition and preprocessing procedures, model architectures, and validation strategies. Consequently, differences in reported accuracy cannot be attributed solely to differences in model architecture.

The strong performance of the proposed model highlights the benefits of integrating spatial and temporal representations of EEG data. The CNN component effectively captured inter-channel relationships and localized spatial patterns, whereas the GRU component modeled long-range temporal dependencies inherent to EEG time series. By combining these complementary representations, the proposed framework provides a more comprehensive characterization of depression-related neural activity than approaches relying primarily on either spatial or temporal information alone. In addition, the incorporation of the mRMR feature selection algorithm reduced feature redundancy and retained the most informative representations, improving computational efficiency and enhancing the model’s generalization performance.

The proposed framework extends existing deep learning approaches for EEG-based depression detection by integrating CNN-based spatial feature extraction, GRU-based temporal modeling, and mRMR feature selection within a unified framework. While previous CNN–GRU models have demonstrated the value of combining spatial and temporal feature learning, the proposed framework further incorporates feature optimization to reduce redundancy and reduce the dimensionality of the fused feature representation while retaining features with high relevance and low redundancy. This integrated design leverages complementary spatial and temporal information together with feature optimization, which likely contributes to the improved classification performance observed in this study.

Several strengths of the present study should be noted. First, the proposed framework integrates complementary spatial and temporal EEG representations through CNN and GRU components rather than relying on either representation alone. Second, mRMR feature selection provides an explicit feature-optimization step that reduces the fused 120-dimensional CNN–GRU representation to 30 informative and nonredundant features before classification. Third, the use of a publicly available dataset improves transparency and facilitates comparison and replication by other researchers. Finally, the framework demonstrates that informative representations for depression classification can be extracted from a limited EEG channel configuration, although further validation is required to determine whether this performance generalizes to independent populations and recording systems.

The high recall and precision observed in the present study indicate that the proposed framework effectively distinguished depression and healthy-control EEG segments within the evaluated dataset. These findings support further investigation of EEG-based deep learning approaches as potential objective adjuncts to conventional depression assessment. However, the present results are based on a relatively small, single-dataset sample and should not be interpreted as evidence of clinical diagnostic performance. Prospective evaluation in larger and independent clinical cohorts will be necessary to determine whether similar performance can be achieved in real-world clinical settings.

Beyond depression classification, EEG-derived features may also have potential applications in treatment research. Noninvasive neuromodulation techniques, such as transcranial direct current stimulation (tDCS) and transcranial magnetic stimulation (TMS), have been used to modulate cortical excitability and neural networks involved in affective and cognitive processing [20,49,50,51,52,53,54,55,56,57,58]. Prior work has also demonstrated effects of noninvasive brain stimulation on neural and behavioral responses, highlighting the potential relevance of individual differences in neural characteristics for understanding variability in stimulation effects [59,60,61,62,63,64]. In this context, future studies could investigate whether EEG-based models such as the framework proposed here may provide complementary biomarkers for monitoring treatment-related neural changes and informing individualized neuromodulation approaches. Such applications were not evaluated in the present study and will require dedicated longitudinal and treatment-focused investigations.

Despite the promising performance of the proposed framework, several limitations should be acknowledged. The present analysis was based on a relatively small sample of 53 participants, including 24 individuals with MDD and 29 healthy controls, from a single publicly available dataset and used EEG recordings acquired with only three electrodes. These factors may constrain the generalizability of the findings. In addition, the model has not yet been externally validated on an independent clinical dataset. Future studies should evaluate the proposed framework in larger and more diverse clinical populations, across independent datasets and recording sites, different EEG acquisition systems and recording protocols, and using high-density EEG recordings. Although the proposed framework reduces the fused CNN–GRU feature representation from 120 to 30 features through mRMR feature selection before classification, the present study did not systematically benchmark hardware-dependent computational measures such as training time, inference latency, and FLOPs/MACs. Therefore, conclusions regarding real-time clinical deployment cannot be drawn from the current results. Future studies should systematically evaluate computational efficiency under standardized hardware configurations and prospective clinical settings to determine the feasibility of real-time implementation.

Overall, the proposed hybrid CNN–GRU–mRMR framework demonstrates promising performance for EEG-based depression classification within the evaluated MODMA dataset. Further validation using larger, independent, and more diverse clinical datasets is necessary to establish its generalizability and potential clinical utility.

### Limitations

The present study did not systematically benchmark hardware-dependent computational measures, and the relatively small sample size (53 participants) from a single dataset limits the generalizability of the findings. Therefore, conclusions regarding clinical applicability should be drawn with caution until further validation on larger and more diverse independent datasets, as well as systematic computational analyses, are performed.

## 6. Conclusions

This study proposed a hybrid deep learning framework that integrates convolutional neural networks, gated recurrent units, and minimum redundancy maximum relevance feature selection for automated depression detection using EEG signals. By jointly modeling the spatial and temporal characteristics of EEG activity while prioritizing informative and non-redundant features, the proposed method demonstrated strong classification performance within the evaluated MODMA dataset.

The findings highlight the value of combining complementary EEG representations for depression classification. The CNN component captures spatial patterns across EEG channels, the GRU models temporal dependencies in the EEG signals, and mRMR reduces redundancy in the fused feature representation. Together, these components provide an effective framework for distinguishing depression and healthy-control EEG segments within the evaluated dataset. Overall, the results support further investigation of hybrid spatial–temporal deep learning approaches for EEG-based characterization of depression.

Future research should validate the framework on larger and more diverse clinical datasets, explore advanced architectures such as attention mechanisms and transformers, integrate multimodal data including neuroimaging and behavioral assessments, and develop interpretability tools to support clinical adoption. Longitudinal studies are needed to assess treatment response tracking, while systematic benchmarking of computational efficiency across hardware platforms and independent external validation are essential to establish generalizability and real-time deployment feasibility.

## Figures and Tables

**Figure 5:**
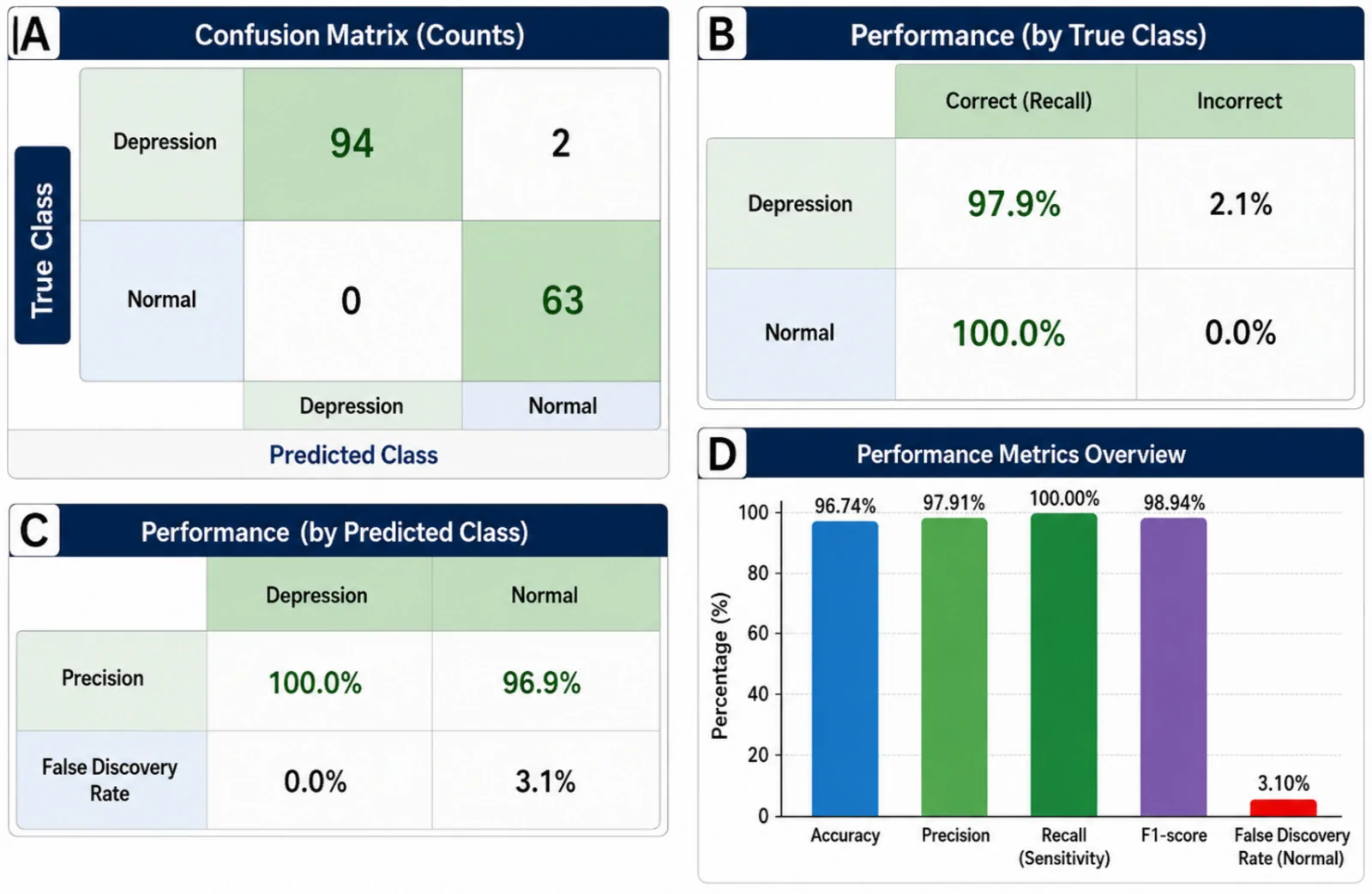
Comprehensive performance evaluation of the proposed EEG-based depression classification framework: (**A**) Confusion matrix showing the numbers of correctly and incorrectly classified depression and healthy control samples. (**B**) Class-wise recall (sensitivity) analysis illustrating the percentage of correctly classified samples for each true class. (**C**) Class-wise precision analysis together with the corresponding false discovery rate (FDR) for each predicted class. (**D**) Overall classification performance of the proposed model in terms of Accuracy, Precision, Recall, and F1-score, demonstrating the effectiveness and robustness of the proposed framework.

**Figure 6:**
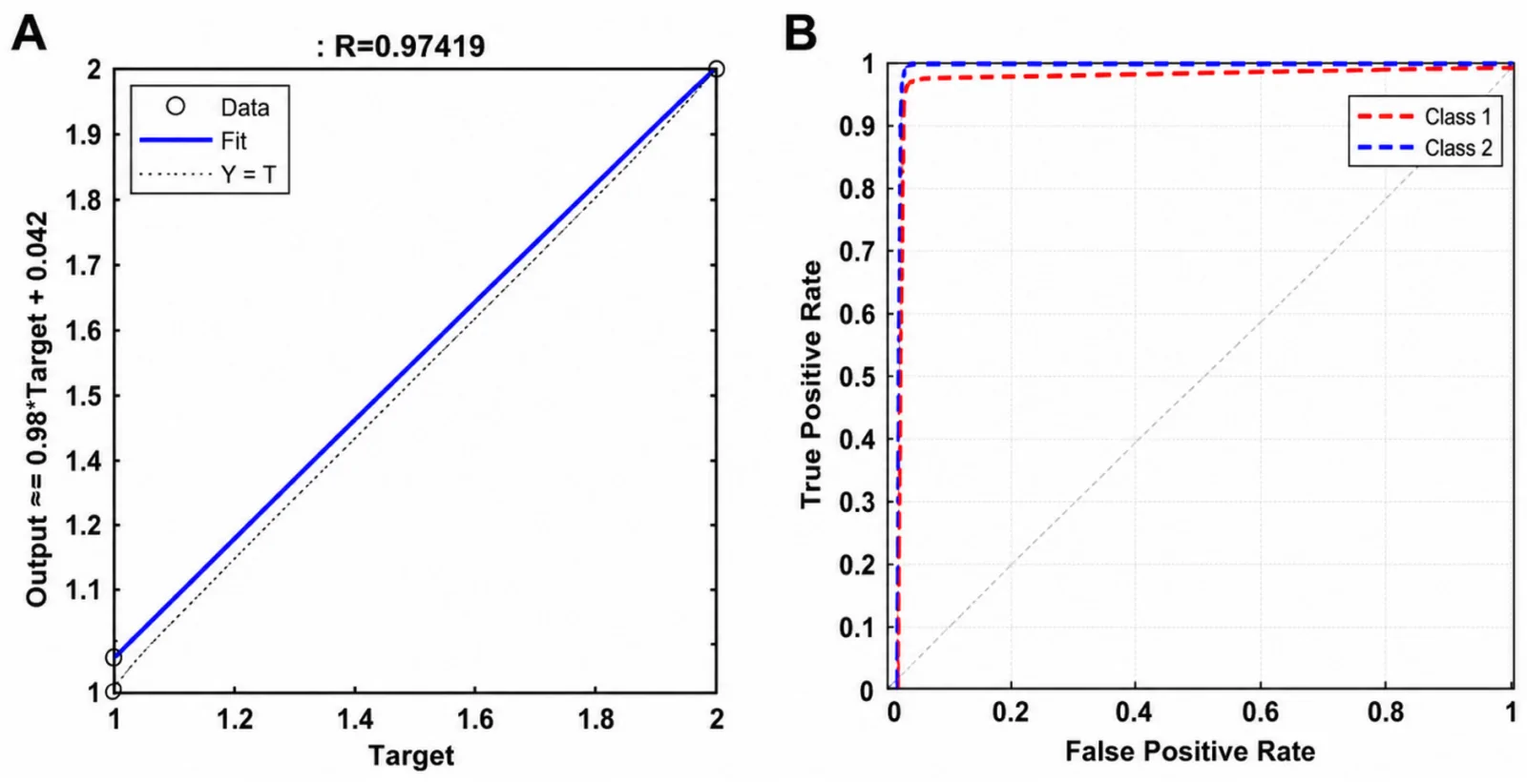
Comprehensive evaluation of the proposed model using regression and receiver operating characteristic (ROC) analyses: (**A**) Regression analysis between predicted and target values, showing the fitted regression line, ideal reference line (Y = T), correlation coefficient (R = 0.97419), and regression equation (Output ≈ 0.98 × Target + 0.042), demonstrating high prediction accuracy and low estimation error. (**B**) Receiver operating characteristic (ROC) curves for depression and healthy control classes, illustrating the classification performance across different decision thresholds. The curves approaching the upper-left corner indicate high sensitivity, low false-positive rates, and excellent discriminative capability of the proposed EEG-based depression classification framework.

**Table 1:** Comparison of the proposed method with other methods.

Method	Accuracy (%)
CNN + GRU [45]	89.63
CNN [46]	91.01
ResNet-50 + LSTM [47]	90.02
CNN [48]	95.00
Proposed Method (CNN-GRU-MRMR-Dense)—Single-Split	96.74
Proposed Method (CNN-GRU-MRMR-Dense)—5-Fold CV	96.74 ± 1.23

## Data Availability

The data presented in this study are openly available in the MODMA dataset at https://modma.lzu.edu.cn/data/application/.
